# Combined Beta-Agonists and Corticosteroids Do Not Inhibit Extracellular Matrix Protein Production *In Vitro*

**DOI:** 10.1155/2012/403059

**Published:** 2012-02-08

**Authors:** Qi Ge, Maree H. Poniris, Lyn M. Moir, Judith L. Black, Janette K. Burgess

**Affiliations:** ^1^Division of Cell Biology, Woolcock Institute of Medical Research, Sydney, P.O. Box M77, Missenden Road, NSW 2050, Australia; ^2^Discipline of Pharmacology, The University of Sydney, Sydney, NSW 2006, Australia

## Abstract

*Background*. Persistent asthma is characterized by airway remodeling. Whereas we have previously shown that neither *β*_2_-agonists nor corticosteroids inhibit extracellular matrix (ECM) protein release from airway smooth muscle (ASM) cells, the effect of their combination is unknown and this forms the rationale for the present study. *Methods*. ASM cells from people with and without asthma were stimulated with TGF*β*1 (1 ng/ml) with or without budesonide (10^−8^ M) and formoterol (10^−10^ and 10^−8^ M), and fibronectin expression and IL-6 release were measured by ELISA. Bronchial rings from nonasthmatic individuals were incubated with TGF*β*1 (1 ng/ml) with or without the drugs, and fibronectin expression was measured using immunohistochemistry. *Results*. Budesonide stimulated fibronectin deposition, in the presence or absence of TGF*β*1, and this was partially reversed by formoterol (10^−8^ M) in both asthmatic and nonasthmatic cells. Budesonide and formoterol in combination failed to inhibit TGF*β*-induced fibronectin in either cell type. A similar pattern of expression of fibronectin was seen in bronchial rings. TGF*β*1-induced IL-6 release was inhibited by the combination of drugs. *Conclusion*. Current combination asthma therapies are unable to prevent or reverse remodeling events regulated by ASM cells.

## 1. Introduction

Airway remodeling, including alterations in the thickness of the basement membrane, an increase in the number of mucus producing cells, an increase in the number of blood vessels (angiogenesis), and a change in the extracellular matrix (ECM) protein profile and hypertrophy/hyperplasia resulting in an increase in the bulk of the airway smooth muscle (ASM), is now recognized as a hallmark feature of asthma. Little is known about the effectiveness of current asthma therapies upon these structural changes in the airways, particularly in the vicinity of the ASM.

We have previously reported that neither corticosteroids nor long-acting *β*_2_-agonists (LABAs) alone are effective at preventing or reversing *in vitro* parameters of ASM-driven airway remodeling [1]. The critical question that remained was whether the combination of these two drug classes would be more effective.

Whilst the combination of inhaled corticosteroids and LABAs improves asthma control and lung function and decreases the frequency of asthma exacerbations compared to placebo or high doses of inhaled corticosteroids alone [2–6], few studies have examined their effectiveness at altering parameters of remodeling *in vivo*. One exception is the study by Orsida and colleagues who reported that the combination of LABAs and inhaled corticosteroids reduces blood vessel number [7]. Given our previous finding of the lack of effectiveness of these drugs singly in reducing parameters of airway remodeling, it was vital to assess their efficacy in combination.

Several studies have examined the *in vitro* effectiveness of combined corticosteroids and LABAs in fibroblasts with conflicting results. Goulet et al. found that corticosteroids and LABAs had opposing effects on matrix protein deposition in the presence of serum and their combination counteracted each other [8]. In contrast, also in fibroblasts, Descalzi et al. reported corticosteroids had significant anti-proliferative effects and that combination with LABAs strengthened these effects [9]. Todorova et al. reported that corticosteroids reduced and the combination with LABAs abolished proteoglycan production induced by serum [10]. In the absence of serum, regardless of whether transforming growth factor *β* (TGF*β*) was present or not, fluticasone increased fibronectin at both the mRNA and protein levels; however, it decreased tenascin-C at both levels. Salmeterol did not affect fibronectin or tenascin-C nor did it alter the effect of fluticasone when the drugs were applied in combination [11].

Whilst we, and others, have begun elucidating the molecular mechanism underlying the synergistic effect of the combination of corticosteroids and LABAs in ASM cells [12, 13], the effect of the combined drugs on the release of ECM proteins from ASM cells remains to be investigated.

In this study, we hypothesized that the combination of corticosteroids and LABAs would be ineffective at inhibiting the production of ECM proteins *in vitro*. To investigate this hypothesis, we examined the effect of the combination of corticosteroids and LABAs in a well-characterized model of *in vitro* airway remodeling [1], namely, TGF**β**-induced fibronectin in human asthmatic and nonasthmatic ASM cells *in vitro* and in nonasthmatic bronchial rings *ex vivo*.

## 2. Materials and Methods

### 2.1. Cell Culture

Approval for all experiments with human lung was provided by the Human Ethics Committees of The University of Sydney and the Sydney South West Area Health Service. Asthmatic ASM was obtained from 7 patients (mean age 32.7 ± 11.5 years SD) either undergoing resection for lung transplantation or deep endobronchial biopsies. Nonasthmatic ASM was obtained from bronchial airways of 9 patients (mean age 58.6 ± 11.6 years SD) undergoing resection for either lung transplantation or carcinoma. The characteristics of the patients are listed in Table 1. Pure ASM bundles were dissected free and grown as explants as previously described [13–15]. ASM cell characteristics were determined by light microscopy and immunofluorescence for the detection of *α*-smooth muscle actin and calponin [16]. All experiments were performed with cells between passages 4 and 8.

### 2.2. Airway Smooth Muscle Cell Treatment

ASM cells from 6 asthmatic and 8 nonasthmatic patients were seeded for 24 hours in 5% fetal bovine serum (FBS) (JRH Biosciences, Melbourne, Australia) Dulbecco's Modified Eagle's Medium (DMEM) (SAFC Biosciences, Lenexa, KS) in the presence of 20 U/mL penicillin, 20 *μ*g/mL streptomycin, and 2.5 *μ*g/mL amphotericin B (Invitrogen, Heidelberg, Australia) at a density of 1 × 10^4^ cells per cm^2^. Medium was then changed to 0.1% insulin transferrin selenium (ITS) (Invitrogen, Heidelberg, Australia) DMEM for 24 hours before addition of formoterol (0.1 and 10 nmol/L) and budesonide (0.1 and 10 nmol/L) alone or in combination as indicated 30 minutes prior to stimulation with TGF*β*1 (1 ng/mL) for the time periods described below. The effect of the drugs in unstimulated cells was assessed by omission of the TGF*β* stimulation in cells maintained in 0.1% ITS. All of the drugs were dissolved in aqueous solutions.

### 2.3. ELISAs

#### 2.3.1. Deposited ECM Protein ELISAs

ASM cells from 6 asthmatic and 6 nonasthmatic patients were seeded in 96 well plates and treated as described above for 48 hours. ECM free of cells was prepared by treatment with sterile hypotonic ammonium hydroxide [17–19]. Fibronectin was measured by ELISA as previously described [19] using an antibody to fibronectin (mouse antihuman plasma fibronectin 2 *μ*g/mL, clone 868A11, Chemicon, Temecula CA) and a purified mouse IgG_1_ isotype control 2 *μ*g/mL, clone MOPC-31C, (Becton and Dickinson Pharmingen, San Jose, CA).

#### 2.3.2. IL-6 ELISAs

ASM cells from 6 asthmatic and 8 nonasthmatic patients were seeded in 24 well plates and treated as described above for 48 hours. Supernatants were collected in aliquots and stored at −20°C until analysis. IL-6 release was detected using an IL-6 ELISA kit according to the manufacturer's instructions (Duoset, Becton and Dickinson, San Jose, CA).

#### 2.3.3. Soluble Fibronectin ELISAs

Supernatants collected as described above were also assayed for soluble fibronectin release using a Quantimatrix Human fibronectin ELISA kit according to the manufacturer's instructions (Chemicon International, Temecula, CA).

#### 2.3.4. Immunohistochemistry

Human lung tissue was obtained from lung specimens resected for carcinoma or transplantation. Bronchial rings (2–5 mm diameter and 3 mm in length) were dissected free from surrounding parenchymal tissue. The bronchial rings were incubated in treatments as described above. After 24 hours, tissues were frozen in optimal cutting temperature (OCT) embedding medium (Fronine Laboratory Supplies, Riverstone, Australia), sectioned on a cryostat and immunohistochemistry performed using mouse anti-fibronectin (1 *μ*g/mL Chemicon International, Temecula, CA) coupled with a horseradish peroxidase labeled polymer. To help identify the morphology of the tissue, hematoxylin and eosin (H&E) staining was performed on adjacent sections. Full details of this method have been described previously [1].

#### 2.3.5. Analysis of Data

For ECM ELISA data, results from duplicate wells from each individual subject were averaged and the absorbance from media alone subtracted before an overall mean and standard error of the mean (SEM) were obtained from asthmatic and nonasthmatic cells. Analysis of variance (ANOVA) repeated measures with bonferonni posttests or student's paired *t*-tests were performed on the results for ECM ELISAs where appropriate. In all cases a *P* value of less than or equal to 0.05 was considered significant.

## 3. Results

### 3.1. Effect of Combined Corticosteroids and LABAs on Basal ECM Protein Deposition

**Figure 1:**
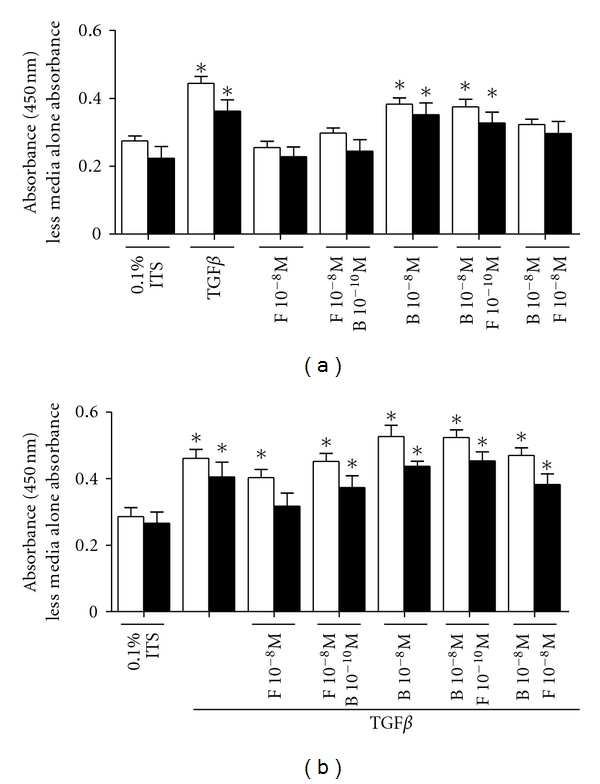
Effect of combined corticosteroids and LABAs on the deposition of fibronectin in the absence (a) or presence of TGF*β* (b) for 48 hrs, respectively. Data are mean ± SEM from *n* = 6 asthmatic (black bars) and nonasthmatic (white bars) ASM cell lines. *Significantly different from nondrug-treated control *P* < 0.05. F: formoterol, B: budesonide.

Budesonide alone (10^−8 ^M) induced fibronectin deposition in both asthmatic and nonasthmatic ASM cells (Figure 1), in agreement with our previous study [1]. The addition of formoterol (10^−8 ^M but not 10^−10 ^M) abolished the induction of fibronectin by budesonide 10^−10 ^M and 10^−8 ^M (Figure 1(a) and Table 2). 

### 3.2. Effect of Combined Corticosteroids and LABAs on TGF*β* Stimulated ECM Protein Deposition

TGF*β* induced the deposition of fibronectin from both asthmatic and nonasthmatic ASM cells, in agreement with our previous reports [1, 20, 21] (Figure 1). The addition of formoterol (10^−10 ^M and 10^−8 ^M) or budesonide (10^−8 ^M or 10^−10 ^M), alone or in combination, did not significantly alter fibronectin deposition in the presence of TGF*β* in either cell type.

### 3.3. Effect of Combined Corticosteroids and LABAs on TGF*β* Stimulated Soluble Fibronectin Release

The release of soluble fibronectin from asthmatic and nonasthmatic ASM cells was increased by TGF*β* but the presence of the drugs, in any combination, did not alter the release of fibronectin (data not shown).

### 3.4. Effect of Combined Corticosteroids and LABAs on Basal IL-6 Release

**Figure 2:**
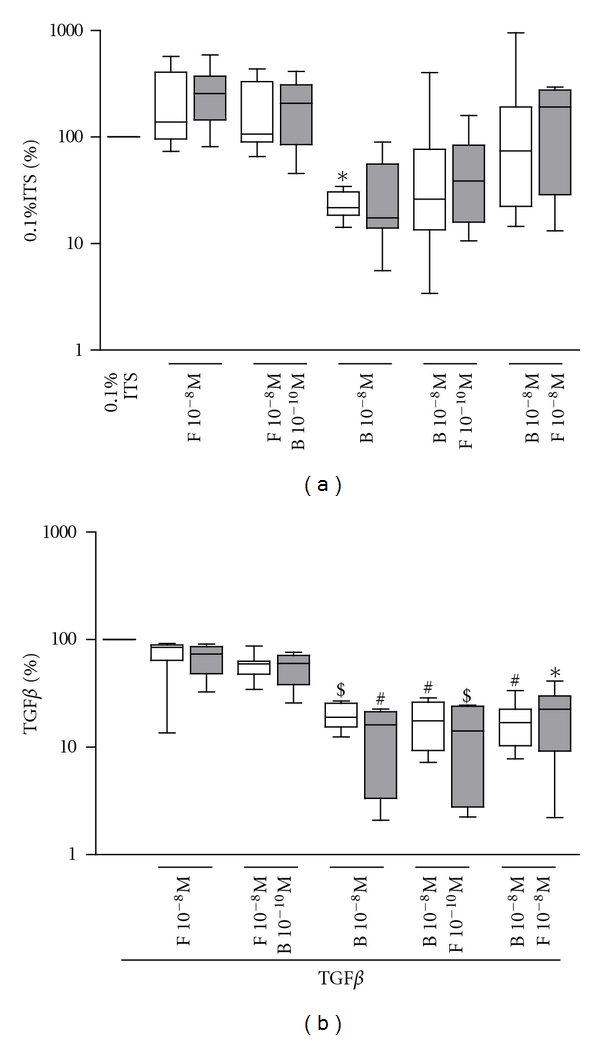
Effect of combined corticosteroids and LABAs on the release of IL-6 in the absence (a) or presence of TGF*β* (b) for 48 hrs respectively. Data are mean ± SEM from *n* = 6 asthmatic (grey boxes) and *n* = 8 nonasthmatic (white boxes) ASM cell lines. Significantly different from nondrug-treated control *P < 0.05, ^#^ *P* < 0.005, ^$^ *P* < 0.001. F: formoterol, B: budesonide.

In nonasthmatic ASM cells, budesonide (10^−8 ^M) alone significantly reduced the release of IL-6. The addition of formoterol (10^−10^ or 10^−8 ^M) did not reverse this reduction (Figure 2(a) and Table 3). The release of IL-6 from asthmatic ASM cells was more variable but followed the same pattern.

### 3.5. Effect of Combined Corticosteroids and LABAs on TGF*β* Stimulated IL-6 Release

In both cell types, TGF*β* significantly induced the release of IL-6. Budesonide reduced the release of IL-6 even in the presence of TGF*β* in both cell types (Figure 2(b) and Table 3). Once again, formoterol (10^−10^ and 10^−8 ^M) did not reverse the inhibitory effect of budesonide (asthmatic 13.66 ± 4.0 and 21.08 ± 5.5, nonasthmatic 17.34 ± 2.9 and 17.55 ± 2.9% of TGF*β*) (Figure 2(b)).

### 3.6. Effect of Combined Corticosteroids and LABAs on Fibronectin Expression in Bronchial Tissue Rings

**Figure 3:**
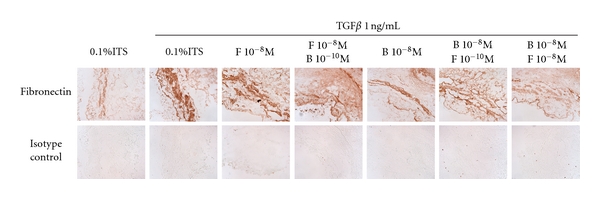
Effect of combined corticosteroids and LABA on TGF*β*-induced fibronectin in nonasthmatic bronchial rings. Immunohistochemical detection of fibronectin (brown staining) basally or following stimulation with TGF*β* in the presence or absence of drugs in nonasthmatic tissue sections.

To examine the effectiveness of the combination of corticosteroids and LABAs on ECM deposition in the whole airway, we used our *ex vivo* bronchial ring model [1, 20]. Bronchial rings from two nonasthmatic individuals stimulated with TGF*β* showed increased deposition of fibronectin, in agreement with our previous findings [1, 20]. Neither formoterol nor budesonide alone, or in combination, reduced the TGF*β*-induced fibronectin deposition (Figure 3).

## 4. Discussion

Our previous work demonstrated that neither long-acting beta agonists nor corticosteroids reduced the release of ECM proteins from ASM cells. The question remained as to whether the combination of these two therapeutic drug classes might be more effective. The results of the current study with formoterol and budesonide demonstrate that this is not the case, regardless of whether the cells were derived from asthmatic or nonasthmatic subjects. Moreover, in bronchial rings stimulated with TGF*β*, fibronectin deposition was not reduced by formoterol, budesonide, nor their combination.

There are many examples of the efficacy of combined LABAs and corticosteroids in both *in vivo* [5, 22] and *in vitro* [13, 23–25] studies. There are very few reports, however, of the modulation of remodeling parameters by these drugs, although the combination of LABAs and inhaled corticosteroids does reduce angiogenesis—one of the features of remodeling [7]. In addition, we have reported a synergistic inhibition of ASM proliferation when these drugs are studied in combination [13]. However, budesonide and salbutamol, alone or in combination, had no effect on collagen fiber tractional remodeling as ASM cells migrated through collagen gels [26]. Descalzi et al. [9] found that the combination of beclomethasone dipropionate (BDP) with either a short or long-acting beta agonist decreased fibronectin production induced by basic fibroblast growth factor, and that this effect was greater than with BDP alone. Their study was carried out in fibroblasts stimulated with basic fibroblast growth factor, as opposed to smooth muscle cells stimulated with TGF*β* in the current study, and this may be the basis for the differences observed. Again, in fibroblasts, corticosteroids in the presence of serum increased ECM deposition, which we also found in ASM cells, but LABAs decreased ECM deposition and the net result of the combination was simply additive [8]. In contrast, Degen et al. found, in fibroblasts, that fluticasone increased fibronectin but decreased tenascin-C mRNA and protein induced by FBS, TGF*β*, or in the absence of stimulation. Under these experimental conditions salmeterol did not influence the fluticasone effects [11]. We also examined the effect of corticosteroids and LABAs alone and in combination on TGF*β*-induced soluble, as opposed to matrix-associated, fibronectin release but again these interventions were without effect in either asthmatic or nonasthmatic cells. To our knowledge, there are no previous reports examining the effect of combination therapy in asthmatic ASM.

A consistent finding from our laboratory has been the increase in release of ECM proteins from ASM in response to corticosteroids. Beclomethasone increased release of fibronectin from ASM [19] and this effect has also been reported by Goulet et al. and Degen et al. in human airway fibroblasts using several corticosteroids [8, 11]. This is consistent with the fact that budesonide increased fibronectin release from ASM cells derived from both asthmatic and nonasthmatic subjects in our current study, and furthermore this occurred whether or not cells were stimulated with TGF*β*. Interestingly, formoterol was able to attenuate budesonide-induced ECM fibronectin deposition even though alone it was without effect. The differential response of fibroblasts to fluticasone in relation to the production of fibronectin and tenascin-C observed by Degen et al. [11] suggests that the individual ECM proteins may respond differently to therapeutic intervention. Therefore, caution should be taken in interpreting the results of this *in vitro* study as a global representation of the effectiveness of current therapies on altering parameters of airway remodeling.

Although we found in the present study that the combination of LABAs and corticosteroids did not decrease fibronectin release, corticosteroids, as previously reported [1, 27], inhibited IL-6 release from the ASM cells. Baouz et al. reported that in (myo) fibroblasts, salmeterol inhibited IL-6 release, and this was amplified by the addition of low concentrations of fluticasone dipropionate [28]. Others have found, also in fibroblasts, that corticosteroids inhibited and LABAs had no effect on IL-6 release and the effect of the combination was that of corticosteroids alone [8]. In contrast, IL-6 release from ASM is increased by *β*_2_-agonists in both asthmatic [1] and nonasthmatic cells [1, 29], and our findings in the current study confirm this.

The study of cells in culture is associated with limitations, and this is where we find the bronchial ring preparation a useful model. It enables us to observe, in an “intact” airway, changes in ECM proteins [1, 17] and cytokine deposition [15, 30] in response to profibrotic stimuli such as TGF*β* and, in addition, to investigate the effects of intervention with relevant therapeutic agents such as LABAs and corticosteroids. Here we confirmed our previous findings [1] that neither LABAs nor corticosteroids alone decreased fibronectin deposition in response to TGF*β* and extended them to include the combination of the two drug classes which were without effect. In contrast, we have previously reported that the phosphodiesterase inhibitor roflumilast abolished TGF*β*-induced fibronectin deposition [1].

In summary, in our cell and tissue models of ECM protein deposition, we investigated whether the combination of a LABA and a corticosteroid would be more effective in inhibiting or reversing TGF*β*-induced fibronectin release. This was not the case either in cells derived from asthmatic or nonasthmatic volunteers, or in intact bronchial rings. Airway remodeling is detrimental in the pathophysiology of asthma, and ECM protein deposition is a major component of said remodeling; therefore, these results highlight the need for further development of agents to reverse or prevent parameters of airway remodeling.

## Figures and Tables

**Table 1:** Patient details.

Patient no.	Age (yrs)	Sex	Disease	Source of tissue	Sample type
1	76	Female	Chronic obstructive pulmonary disease	Endobronchial biopsy	ASM
2	43	Female	Asthma	Endobronchial biopsy	ASM
3	26	Male	Asthma	Endobronchial biopsy	ASM
4	66	Male	Chronic obstructive pulmonary disease	Resection	ASM
5	22	Male	Asthma	Endobronchial biopsy	ASM
6	38	Female	Carcinoid (atypical)	Resection	ASM
7	46	Female	Carcinoma	Resection	ASM
8	58	Male	Emphysema	Explanted lungs	ASM
9	50	Female	Asthma	Endobronchial biopsy	ASM
10	56	Female	Emphysema	Explanted lungs	ASM
11	40	Male	Asthma	Endobronchial biopsy	ASM
12	27	Male	Asthma	Endobronchial biopsy	ASM
13	68	Female	Carcinoma	Resection	ASM
14	55	Male	Emphysema	Explanted lungs	ASM
15	64	Female	Emphysema	Explanted lungs	ASM
16	21	Male	Asthma	Endobronchial biopsy	ASM
17	25	Female	Bronchiolitis Obliterans	Explanted lungs	Bronchial rings
18	48	Female	Emphysema	Explanted lungs	Bronchial rings

ASM: airway smooth muscle.

**Table 2:** Effect of combined corticosteroids and LABAs on basal and TGF*β*-stimulated ECM protein deposition.

Asthmatic		Alone			Nonasthmatic	Alone		
		—	F 10^−10 ^M	F 10^−8 ^M		—	F 10^−10 ^M	F 10^−8 ^M

Alone	—			104.8 ± 6.7	—			92.3 ± 2.5
	B 10^−10 ^M			111.5 ± 9.2	B 10^−10 ^M			108.5 ± 2.1
	B 10^−8 ^M	166.4 ± 13.4*	154.8 ± 13.0	137.1 ± 8.3	B 10^−8 ^M	140.5 ± 7.4*	137.3 ± 7.7	117.9 ± 4.4

		TGF*β* stimulated				TGF*β* stimulated		
		—	F 10^−10 ^M	F 10^−8 ^M		—	F 10^−10 ^M	F 10^−8 ^M

TGF*β* stimulated	—			78.3 ± 2.5*	—			87.4 ± 2.0*
	B 10^−10 ^M			93.6 ± 3.8	B 10^−10 ^M			98.5 ± 3.6
	B 10^−8 ^M	113.5 ± 10.2	116.0 ± 8.1	97.1 ± 6.5	B 10^−8 ^M	114.1 ± 3.0*	114.2 ± 2.8	102.3 ± 2.7

F: formoterol, B: budesonide.

Data are expressed as % 0.1% ITS for drugs alone and % TGF*β* for TGF*β*-stimulated samples.

*significantly diff to 0.1% ITS or TGF*β* *P* < 0.05. *n* = 6 asthmatic and 6 nonasthmatic.

**Table 3:** Effect of combined corticosteroids and LABAs on basal and TGF*β*-stimulated IL6 release.

Asthmatic		Alone			Nonasthmatic	Alone		
		—	F 10^−10 ^M	F 10^−8 ^M		—	F 10^−10 ^M	F 10^−8 ^M

Alone	—			275.6 ± 71.4	—			224.0 ± 68.2
	B 10^−10 ^M			208.3 ± 54.8	B 10^−10 ^M			181.5 ± 51.6
	B 10^−8 ^M	31.9 ± 12.7	54.1 ± 22.5	166.6 ± 48.7	B 10^−8 ^M	23.35 ± 2.5	78.13 ± 47.5	187.1 ± 112.7

		TGF*β* stimulated				TGF*β* stimulated		
		—	F 10^-10 ^M	F 10^-8 ^M		—	F 10^−10 ^M	F 10^−8 ^M

TGF*β* stimulated	—			68.2 ± 9.2	—			74.1 ± 9.5
	B 10^−10 ^M			55.8 ± 7.9	B 10^−10 ^M			58.78 ± 5.4
	B 10^−8 ^M	13.7 ± 3.6	13.7 ± 4.0	21.1 ± 5.5	B 10^−8 ^M	20.0 ± 1.9	17.3 ± 2.9	17.6 ± 2.9

F: formoterol, B: budesonide.

Data are expressed as mean ±SEM % 0.1% ITS for drugs alone and % TGF*β* for TGF*β*-stimulated samples. *n* = 6 asthmatic and 8 nonasthmatic.
